# The central neurometabolic fingerprint of persistent spinal pain syndrome type 2: A prospective [^18^F]FDG PET/CT neuroimaging study

**DOI:** 10.1016/j.ynpai.2026.100235

**Published:** 2026-10-09

**Authors:** Lara S. Burmeister, Max van Grinsven, Bert-Kristian van Roosendaal, Richard L. Witkam, Inge Arnts, Stephanie Janssen, Jessica Wegener, Kris C.P. Vissers, Anne I.J. Arens, Martin Gotthardt, Michael Rullmann, Dylan Henssen

**Affiliations:** a Department of Medical Imaging, Radboud University Medical Center, Nijmegen, Nijmegen, 6525GA, The Netherlands; b Department of Anesthesiology, Pain and Palliative Medicine, Radboud University Medical Center, Nijmegen, 6525GA, the Netherlands; c Chronic Pain Department, Sint Maartenskliniek, Nijmegen, 6574NA, the Netherlands; d Department of Nuclear Medicine, University Hospital Leipzig, Leipzig, 04103, Germany

**Keywords:** [^18^F]FDG PET/CT, Brain metabolism, Persistent spinal pain syndrome type 2

## Abstract

Persistent spinal pain syndrome type 2 (PSPS-T2) is a disabling pain condition that persists after spinal surgery. While spinal cord stimulation (SCS) offers symptomatic relief, objective biomarkers to guide diagnosis and treatment are lacking.

In this prospective cohort study, fifteen PSPS-T2 patients underwent dedicated [^18^F]FDG PET/CT brain imaging prior to SCS implantation. Group-level metabolic patterns were compared to matched healthy controls using statistical parametric mapping. Regional metabolism was correlated with pain scores and neurotransmitter PET atlases using JuSpace.

PSPS-T2 patients exhibited a distributed pattern of altered glucose metabolism involving frontal, temporal and cerebellar regions. Significant hypermetabolism was observed in the left temporal pole, right inferior temporal gyrus, and bilateral cerebellar lobule VIII, whereas hypometabolism was identified in the right fusiform gyrus, right superior frontal gyrus, left gyrus rectus, and cerebellar vermis. Moreover, there were significant spatial correlations between these metabolic changes and serotonergic and glutamatergic neurotransmitter systems. Furthermore, higher baseline pain was associated with increased frontal and temporal metabolism, whereas post-intervention pain was negatively correlated with metabolism in the temporal pole.

These findings suggests that PSPS-T2 is characterized by network-level alterations in brain regions integrating sensory, emotional, and cognitive aspects of pain, with a neurochemical signature implicating serotonergic and glutamatergic systems. Although preliminary, this works provides evidence for a disease-relevant neurometabolic profile and highlights the potential of [^18^F]FDG PET/CT for investigating central pain mechanisms. Future research should focus on validating this marker in larger populations.

## Highlights

• PSPS-T2 patients show altered brain metabolism before SCS implantation.
• Temporal and frontal metabolism links to baseline and post-treatment pain intensity.
• Metabolic patterns align with serotonergic and glutamatergic systems.
• Findings support network-level dysregulation of pain processing.

## 1 Introduction

Chronic back pain is a global health concern that affects people of all ages and is recognized as a leading contributor to disability-adjusted life years worldwide (Duthey, 2013). A common cause of chronic back pain is the so-called post-surgical persistent spinal pain syndrome (PSPS-T2) (Waguespack et al., 2002). PSPS-T2 thereby refers to chronic axial pain and/or extremity pain, that persists or recurs after spinal surgery (Christelis et al., 2021). It is estimated that up to 40% of patients who have undergone spinal surgery suffer from persistent or recurrent pain (Thomson, 2013). Despite its prevalence and significant burden, the aetiology of PSPS-T2 remains largely elusive and there are currently no pathognomonic characteristics of this condition (Durand et al., 2015; el Barzouhi et al., 2013; Sebaaly et al., 2018). The diagnostic work-up for PSPS-T2 requires excluding serious underlying causes of pain such as infection or malignancy, with the goal of identifying the most probable aetiology of the spinal lesion (van de Minkelis et al., 2024). Albeit, no correlation has been found between morphological changes after spinal surgery and patient's symptoms. Additionally, diagnostic imaging studies of the lumbar region only produced non-specific results (Baronio et al., 2020; Witkam et al., 2022). Hence, there is an urgent need for validated diagnostic biomarkers of PSPS-T2 and chronic pain in general (Tracey et al., 2019). In the quest for neuroimaging biomarkers in chronic pain patients, changes in functional activity of a wide variety of brain regions have been described (van der Miesen et al., 2019; Wager et al., 2013). However, molecular imaging methods could enable the in vivo evaluation of neurometabolic changes and neurochemical networks and their specific receptors (Maarrawi et al., 2007).

In this study, we aimed to investigate the neurometabolic footprint of PSPS-T2 using a dedicated 2-[^18^F]fluoro-2-deoxy-d-glucose ([^18^F]FDG) neuroimaging protocol, thereby contributing to the search for objective molecular imaging biomarkers and further elucidating the pathophysiology for this condition. The primary goal was to characterize brain glucose metabolism in PSPS-T2 patients prior to initiation of neuromodulation therapy, in this case spinal cord stimulation (SCS). SCS is a form of neuromodulation for treating patients with PSPS-T2 when conservative pain management and interventional pain procedures fail to deliver adequate pain relief (Kumar et al., 2007). In addition to our primary analysis, we also explored whether [^18^F]FDG patterns assessed at baseline could be associated with clinical outcomes following SCS therapy, thereby providing first insights into potential associative biomarkers for neuromodulation response.

## 2 Materials and methods

### 2.1 Participants and SCS procedure

Patients aged 18 years or older diagnosed with PSPS-T2 who were on the waiting list to receive neuromodulation therapy (i.e., SCS implantation) were included in this study. Criteria for exclusion were any known contraindications for PET imaging and the co-existence of other neurological disorders with potential cerebral localization, including movement disorders, mild cognitive impairment and any form of dementias. Furthermore, participants with other disabling chronic pain conditions or diabetes mellitus were excluded, as these factors could compromise the validity of PET/CT imaging and affect the outcomes of SCS treatment. As part of the standard pre-implantation assessment for SCS, patients underwent psychological screening for factors that could interfere with SCS treatment. Patients with psychological comorbidities considered incompatible with SCS treatment were excluded from SCS implantation and were thus also not included in the present study. To screen for any undiagnosed cognitive impairment at the time of PET imaging, all patients underwent the Mini Mental State Examination (MMSE). To assess pain levels, an 11-point numerical rating scale (NRS) ranging from 0 to 10 with 10 indicating the highest pain level, was used at baseline and 3 months post-SCS implantation. At each assessment, patients were asked to report their average pain intensity during the week preceding the respective assessment time point. PET/CT findings were compared with those of 20 healthy, pain-free controls (mean age 62.2 ± 9.4 years; 9 females). The control cohort consisted of adults without pain with visually and quantitatively normal cerebral metabolism on [^18^F]FDG PET and has been described in detail elsewhere (Loewenstein et al., 2024).

The SCS systems were implanted through a standardized and widely used procedure, meaning that the electrodes were placed percutaneously and eventually connected to an internalized implantable pulse generator in either the inferior abdominal or gluteal region. Appropriate positioning of the electrode was checked for by delivering tonic stimulation during the implantation procedure, of which the coverage had to be at least ≥80% of the painful area. After SCS implantation, the patients were followed for a follow-up period of at least three months.

### 2.2 PET image acquisition and post-processing

All imaging data were acquired on a hybrid PET/CT system (Biograph Vision, Siemens Healthineers, Erlangen, Germany). Following the SNMMI Procedure Standard (Arbizu et al., 2024) and the EANM Practice Guideline (Guedj et al., 2022), blood glucose levels were checked prior to [^18^F]FDG PET imaging in order to avoid imaging of hyperglycaemic patients (defined as blood glucose levels >160 mg/ dl; >8.9 mmol/L). Thirty minutes post-injection of a mean intravenous bolus injection of 150 MBq [^18^F]FDG, static brain PET data were acquired in 3D list-mode over 20 min and reconstructed into a 256 × 256 matrix (voxel size: 1.00 × 1.00 × 2.03 mm^3^) employing the built-in ordered subset expectation maximization algorithm with 8 iterations, 21 subsets and a 3 mm Gaussian filter. For attenuation correction, a low dose CT scan was made. Post-processing consisted of image reorientation in a canthocaudal orientation and attenuation of uptake. Images were displayed in standard grey-scale in three anatomical planes (axial, coronal and sagittal). These reconstructions were used for visual assessment by two nuclear medicine physicians (A.A. and D.H.) to exclude other pathological conditions.

### 2.3 Statistical parametric mapping

The PET images were spatially normalized based on a MNI template (MNI152) within the SPM12 software (Statistical Parametric Mapping, Wellcome Department of Imaging Neuroscience Group, London, UK; http://www.fil.ion.ucl.ac.uk/spm). Regional tracer uptake was intensity-normalized to whole-brain activity, expressed as standardized uptake value ratios (SUVRs). For spatial smoothing, a standard 8-mm full-width at half-maximum Gaussian kernel was applied. The spatially normalized and smoothed PET data were entered into a group-level two-sampled *t*-test within SPM12 to test for voxel-wise differences between PSPS-T2 patients and non-painful controls. Voxel-wise statistical significance was assessed using false-discovery rate (FDR) correction and an exploratory uncorrected threshold of *p* < 0.001 with a minimum cluster size of at least 100 contiguous voxels were also conducted. Additional exploratory maps are provided.

### 2.4 Spatial correlation with neurotransmitter maps

The JuSpace toolbox (version 2.0) (*https://github.com/juryxy/JuSpace/tree/JuSpace_v2.0*) enables the spatial correlation of different imaging modalities with neurotransmitter atlases derived from molecular imaging techniques (i.e., PET and single-photon-emission computed tomography (SPECT) studies). The [^18^F]FDG PET data were z-scored relative to controls and parcellated into 119 regions delineated by the Neuromorphometrics atlas, containing different neurotransmitter atlases. The neurotransmitter atlases used in this study were: 5-hydroxytryptamine receptor subtypes 1a (5HT1a), 1b (5HT1b), 2a (5HT2a), 4 (5HT4) (Beliveau et al., 2017) and 6 (5HT6) (Radhakrishnan et al., 2018); α4β2 nicotinic acetylcholine receptor (A4B2) (Hillmer et al., 2016); cannabinoid receptor 1 (CB1) (Laurikainen et al., 2019); cyclooxygenase-1 (COX1) (Kim et al., 2020); dopamine receptor subtypes 1 (D1) (Kaller et al., 2017) and 2 (D2) (Alakurtti et al., 2015); dopamine transporter (DAT) (Dukart et al., 2018); fluorodopa (FDOPA)(García-Gómez et al., 2018); gamma-aminobutyric acid type a receptor (GABAa) (Dukart et al., 2018); kappa opioid receptor (KOR) (Vijay et al., 2018); mu-opioid receptor (MOR) (Turtonen et al., 2021); translocator protein expression (Microglia) (Lois et al., 2018); *N*-methyl-d-aspartate receptor (NMDA) (Galovic et al., 2021); serotonin transporter (SERT) (Fazio et al., 2016); synaptic vesicle glycoprotein 2 A (SV2A) (Finnema et al., 2018); and metabotropic glutamate receptor 5 (mGluR5) (Hansen et al., 2022). Spearman correlations were computed to investigate correlations between the [^18^F]FDG PET data and the aforementioned neurotransmitter atlases. This analysis was adjusted for spatial autocorrelation using the grey matter tissue probability map from SPM12. Exact permutation-based *p*-values were calculated using 10,000 permutations and were corrected for multiple comparisons using the false-discovery rate (FDR) method, setting the significance at *p* < 0.05. This statistical threshold was chosen in accordance with the original JuSpace framework and prior applications of this method, where FDR correction is used to control for multiple comparisons across molecular PET atlases (see (Dukart et al., 2021)). FDR correction applied using the Benjamini–Hochberg procedure across 1024 correlation analyses. Results were considered significant at q < 0.05.

### 2.5 Statistical analysis

Statistical analyses were performed using SPSS (IBM Corporation, Version 29.0.2.0). To explore regional brain–pain associations, Pearson correlation coefficients were calculated between standardized uptake value ratio (SUVR) values of individual brain regions and clinical pain measures (Numerical rating scale (NRS) at baseline, and percentual difference in NRS score between baseline and 3 months post-SCS implantation (%)). Subject age was taken as a covariate in these analyses as cerebral metabolism is known to decline with age. SUVR values were extracted from 173 AAL3-defined regions. All variables were tested for normality and visually inspected for outliers. Correlation analyses were restricted to the predefined pain-related outcomes. Resulting *P*-values were corrected for multiple comparisons using the false discovery rate (FDR; Benjamini-Hochberg procedure). Statistical significance was defined as *P* < 0.05, FDR-corrected.

## 3 Results

In total, 15 PSPS-T2 patients (mean age 55 ± 9.4 years; 9 females) were included in this study. No significant differences in mean age were found between patients and subjects in the control group (*F*(2, 54) = 0.04, *p* = 0.96). Mean MMSE score of the PSPS-T2 patients was found to be 29.7 ± 0.6 points; no patients scored lower than 28 points out of a maximum of 30 points, which indicated no evidence of mild cognitive impairment. An overview of the PSPS-T2 patients and the control cohort can be found in Table 1. All patients adhered to both the imaging protocol and the follow-up period, resulting in no missing data. Visual assessment of the images found no neurodegenerative PET patterns or other features of intracranial pathology. The average pain intensity at baseline was 7.3 ± 1.03 points on the 11-point NRS scale. All patients received SCS implantation. No adverse events were reported. Three months post-SCS implantation, the relative pain reduction across patients, based on their predominant pain complaint at baseline, was on average 69.2 ± 10.9%.

**Table 1:** Demographics and image-related characteristics.

Variable	PSPS-T2 patients (*n* = 15)	Local controls (*n* = 20)	*p*-value
Age, years (mean ± SD)	55.0 ± 9.4	62.2 ± 9.4	0.96
Sex, female/male	9 / 6	9 / 11	N/A
BMI, kg/m^2^ (mean ± SD)	28 ± 3	27 ± 3	N/A
MMSE (mean ± SD)	29.7 ± 0.6	N/A	N/A
Pain at baseline (mean ± SD)	7.3 ± 1.03	N/A	N/A

### 3.1 Regional alterations in cerebral glucose metabolism in PSPS-T2 patients

Compared to healthy, pain-free controls, PSPS-T2 patients showed regional alterations in cerebral glucose metabolism, including both hyper- and hypometabolic clusters at exploratory thresholds. Thereby, hypermetabolism surviving correction for multiple comparison at the cluster level (FDR-corrected *p* < 0.05) was observed in the left temporal pole and right inferior temporal gyrus, as well as in bilateral cerebellar lobule VIII. Additional hypermetabolic clusters were found in the left cerebellar Crus II, insula, supramarginal gyrus, Rolandic operculum, lingual gyrus, medial orbitofrontal cortex, and locus coeruleus. Since these clusters did not survive correction of multiple comparison, these findings should be considered exploratory (Table 2).

**Table 2:** Brain regions that show hypermetabolism in PSPS-T2 patients as compared to non-painful, healthy controls.

x[mm]	y [mm]	z [mm]	Area	Brodmann area	FDR corrected p-value
44	−52	−54	Right cerebellar lobule VIII	N/A	0.007*
−32	2	−44	Left middle temporal pole	38	< 0.001*
−40	−58	−58	Left cerebellar lobule VIII	N/A	0.019*
52	−6	−40	Right inferior temporal gyrus	20	0.001*
4	−26	−42	Right locus coeruleus	N/A	0.114
−14	−82	−46	Left cerebellar Crus II	N/A	0.528
18	−100	−12	Right lingual gyrus	18	0.528
−60	−28	44	Right supramarginal gyrus	N/A	0.528
32	24	2	Right insula	13	0.537
−38	−30	16	Left Rolandic operculum	40	0.632
−18	−98	−18	Left lingual gyrus	18	0.528
0	36	−12	Left medial orbitofrontal cortex	11	0.632

Results of SPM analyses of [^18^F]FDG PET data displayed as MNI coordinates and corresponding brain areas (according to automated anatomical labelling atlas) and Brodmann areas. Asterisks indicate clusters (and their local maxima) that survived FDR correction at *p* < 0.05.

Regional hypometabolism was also observed in PSPS-T2 patients relative to controls (Table 3, Fig. 1). These patterns mainly included occipital-temporal and frontal regions, with significant clusters (FDR-corrected *p* < 0.05) in the right fusiform gyrus, right superior frontal gyrus, and left gyrus rectus. Additionally, a significant cluster was found in the cerebellar vermis (lobule III). Moreover, there was hypometabolism in cerebellar regions such as the cerebellar lobule X and the right cerebellar Crus II. Other regions involved the precentral gyrus and the globus pallidus. However, none of the clusters survived correction of multiple comparison at the cluster level (FDR-corrected *p* < 0.05), and therefore these findings should be considered exploratory as well.

**Table 3:** Brain regions that show hypometabolism in PSPS-T2 patients as compared to non-painful, healthy controls.

x [mm]	y [mm]	z [mm]	Area	Brodmann area	FDR corrected p-value
−32	−78	−2	Right fusiform gyrus	18	<0.001*
18	48	−8	Right superior frontal gyrus, part 2	N/A	0.011*
44	−38	−12	Right fusiform gyrus	N/A	0.001*
−18	22	−12	Left gyrus rectus	N/A	0.005*
−30	−26	−42	Left cerebellar lobule X	N/A	0.531
0	−94	−26	Right cerebellar Crus II	N/A	0.103
32	−24	−42	Right cerebellar lobule X	N/A	0.531
−48	−6	20	Left precentral gyrus	6	0.103
2	−40	−2	Vermis lobule III	N/A	0.042*
−12	2	2	Left globus pallidus	N/A	0.248

Results of SPM analyses of [^18^F]FDG PET data displayed as MNI coordinates and corresponding brain areas (according to automated anatomical labelling atlas) and Brodmann areas. Asterisks indicate clusters (and their local maxima) that survived FDR correction at *p* < 0.05.

**Fig. 1:**
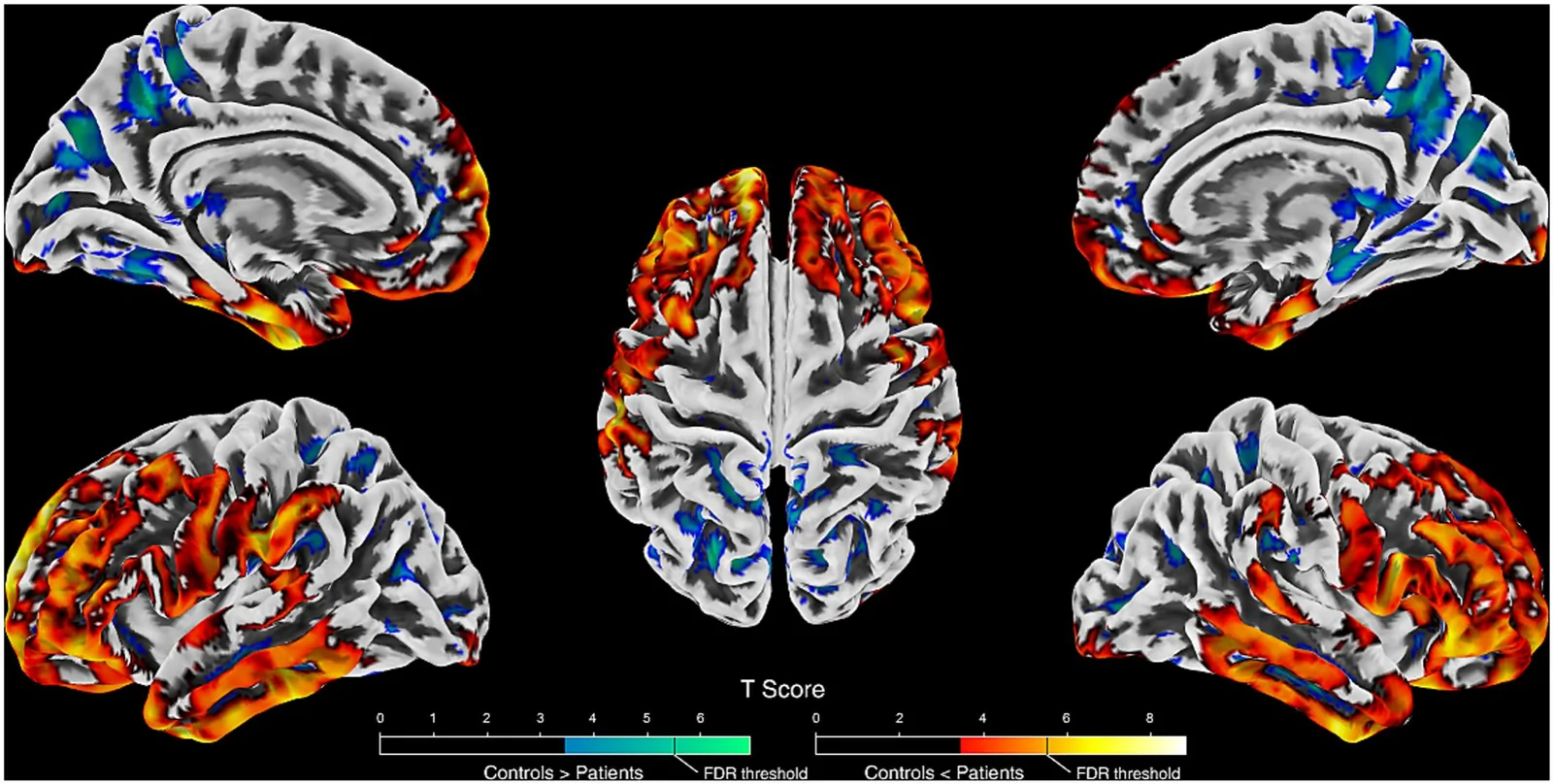
Clusters of significant hypermetabolism (in red) and hypometabolism (in blue) in PSPS-T2 patients as compared to control subjects without pain. Three-dimensional surface projections of [^18^F]FDG PET results in PSPS-T2 patients compared to pain-free controls. Regions of relative hypermetabolism are shown in red-yellow, and involve predominantly temporal and frontal cortical regions, including areas such as the middle temporal pole, inferior temporal gyrus, medial orbitofrontal cortex. Areas of hypometabolism are shown in blue-green, with involvement of frontal and temporal, parietal and subcortical regions, including the superior frontal gyrus, gyrus rectus, fusiform gyrus, precentral gyrus, globus pallidus, cerebellar lobule X, cerebellar crus II, and vermis lobule III. Renderings are displayed as medial and lateral views of the left and right hemispheres, together with a superior view of the cerebral cortex. Colors represent T-scores as indicated by the color bars (i.e., red-yellow for hypermetabolism and blue-green for hypometabolism). The FDR-corrected threshold is indicated on the color bar. (For interpretation of the references to color in this figure legend, the reader is referred to the web version of this article.)

### 3.2 Alignment of neurometabolic changes with neurotransmitter systems

In a spatial correlation analysis between regional brain metabolism derived from [^18^F]FDG PET imaging in this study and publicly available neurotransmitter PET maps, significant associations were observed across multiple molecular systems (Fig. 2). First, the spatial distribution of *Z*-scores of the regional brain metabolism maps correlated positively with the regional availability of serotonergic receptors including 5-HT1a (*FDR-corrected p = 0.0029*), 5-HT1b (*FDR-corrected p = 0.0018*), 5-HT2a (*FDR-corrected p = 0.0002*), and 5-HT4 (*FDR-corrected p = 0.0035*), as well as with mGluR5 (*FDR-corrected p = 0.0002*). Second, the Z-scores of regional brain metabolism maps showed a significant negative correlation with 5-HT6 *FDR-corrected p = 0.0035*), A4B2 nicotine receptors (*FDR-corrected p = 0.0002*), as well as with dopaminergic markers, including D2 (*FDR-corrected p = 0.0022*), DAT (*FDR-corrected p = 0.0338*), and [^18^F]FDOPA uptake maps (*FDR-corrected p = 0.0002)*. Furthermore, there were significant negative correlations with cannabinoid CB1 (*FDR-corrected p = 0.0002*), kappa opioid receptors (*FDR-corrected p = 0.0004*), NMDA receptors (*FDR-corrected p = 0.0002*) and COX-1 (*FDR-corrected p = 0.0002*). Lastly, there were significant negative correlations with microglia (*FDR-corrected p = 0.002*), synaptic vesicle glycoprotein 2a (*FDR-corrected p = 0,0022*) and serotonin transporters (*FDR-corrected p = 0.0004*). No significant associations were observed for D1, GABAa, or mu-opioid receptor (MOR) maps (see Table 4).

**Fig. 2:**
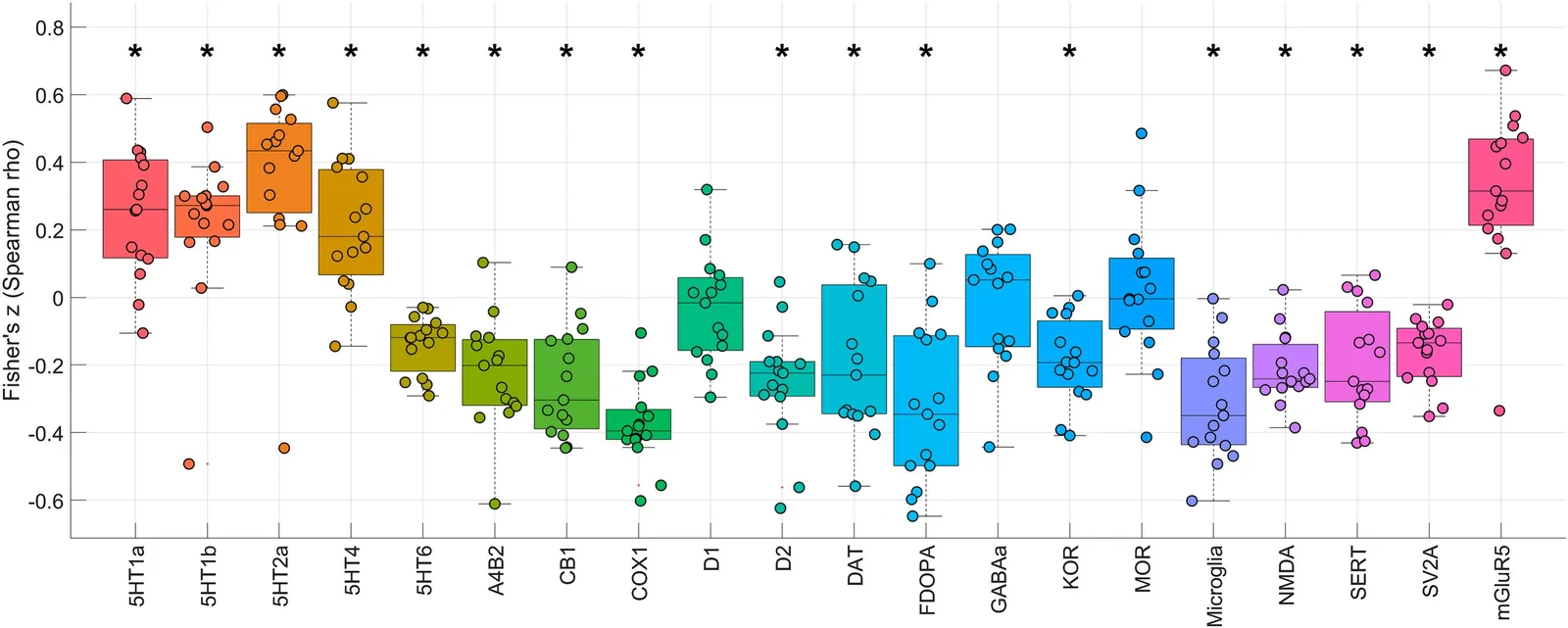
Spatial correlations between regional brain metabolism and molecular PET maps. Bar plot showing Fisher's *z*-transformed Spearman correlation coefficients between regional [^18^F]FDG PET *Z*-score maps and 20 publicly available molecular PET receptor density maps. Each coloured bar represents the group-level spatial correlation between the FDG z-scores and a specific PET target. Dots represent individual subject-level spatial correlation coefficients. Error bars denote the 95% confidence interval of the group mean. Significant associations after FDR correction are indicated with asterisks (*p < 0.05; **p < 0.01). Positive correlations were observed with serotonergic receptors (5-HT1a, 5-HT1b, 5-HT2a, 5-HT4) as well we with mGluR5 suggesting involvement of both neurotransmitter systems in the metabolic alterations observed in PSPS-T2. Negative correlation were found with 5-HT6, A4B2 nicotine receptors, as well as dopaminergic markers (D2, DAT and [^18^F]FDOPA uptake maps). Furthermore, there were significant negative correlations with cannabinoid CB1, kappa opioid receptors, NMDA receptors, COX-1, microglia, synaptic vesicle glycoprotein 2a and serotonin transporters. No significant associations were observed for D1, GABAa, or mu-opioid receptor (MOR) maps. 5HT = serotonin receptor (5HT1a, 5HT1b, 5HT2a, 5HT4 and 5HT6); A4B2 = α4β2 nicotinic acetylcholine receptor; CB1 = cannabinoid receptor type 1; COX1 = cyclooxygenase-1; D1/D2 = dopamine D1/D2 receptor; DAT = dopamine transporter; FDOPA = fluorodopa uptake; GABAa = γ-aminobutyric acid A receptor; KOR = kappa-opioid receptor; MOR = mu-opioid receptor; Microglia = translocator protein expression; NMDA = *N*-methyl-d-aspartate receptor; SERT = serotonin transporter; SV2 A = synaptic vesicle glycoprotein 2 A; mGluR5 = metabotropic glutamate receptor subtype 5.

**Table 4:** Spatial correlation between regional brain metabolism maps in PSPS-T2 patients and publicly available neurotransmitter PET maps.

PET Map	Mean Fisher's Z (Spearman rho)	p-value	FDR corrected p-value
5-HT1a	0.249	0.002	0.0029*
5-HT1b	0.214	0.001	0.0018*
5-HT2a	0.362	1e-04	0.0002*
5-HT4	0.209	0.003	0.0035*
5-HT6	−0.138	0.003	0.0035*
A4B2	−0.226	0.0001	0.0002*
CB1	−0.251	0.0001	0.0002*
COX1	−0.377	0.0001	0.0002*
D1	−0.035	0.600	0.6664
D2	−0.253	0.001	0.0022*
DAT	−0.187	0.029	0.0338*
FDOPA	−0.325	0.0001	0.0002*
GABAa	−0.014	0.861	0.8610
KOR	−0.188	0.0002	0.0004*
MOR	0.021	0.809	0.8520
Microglia	−0.315	0.0001	0.0002*
NMDA	−0.211	0.0001	0.0002*
SERT	−0.198	0.0002	0.0004*
SV2A	−0.162	0.001	0.0022*
mGluR5	0.318	0.0001	0.0002*

Spatial correlations between regional brain metabolism patterns observed in PSPS-T2 patients (Z-scored [^18^F]FDG PET maps from this study) and neurotransmitter distribution maps derived from publicly available PET atlases. Reported are Spearman's rank correlation coefficients (Fisher's Z), uncorrected *p*-values, and false discovery rate (FDR)–corrected *p*-values. Significant associations (FDR-corrected *p* < 0.05) are indicated with an asterisk (*). Metabolic changes showed a positive spatial correlation with the 5-HT1a, 5-HT1b, 5-HT2a, 5-HT4 and mGluR5 receptor maps and a negative correlation with 5-HT6, A4B2, CB1, COX1, D2, DAT, FDOPA, KOR, Microglia, NMDA, SERT and SV2A maps suggesting potential involvement of serotonergic and dopaminergic systems in the pathophysiology of PSPS-T2.

5HT = serotonin receptor (5-HT1a, 5-HT1b, 5-HT2a, 5-HT4 and 5-HT6); A4B2 = α4β2 nicotinic acetylcholine receptor; CB1 = cannabinoid receptor type 1; COX1 = cyclooxygenase-1; D1/D2 = dopamine D1/D2 receptor; DAT = dopamine transporter; FDOPA = fluorodopa uptake; GABAa = γ-aminobutyric acid A receptor; KOR = kappa-opioid receptor; MOR = mu-opioid receptor; Microglia = translocator protein expression; NMDA = *N*-methyl-d-aspartate receptor; SERT = serotonin transporter; SV2A = synaptic vesicle glycoprotein 2A; mGluR5 = metabotropic glutamate receptor subtype 5.

### 3.3 Correlation of metabolic changes with pain intensity

Significant positive correlations were found between baseline pain intensity scores and SUVR-value in the right superior frontal gyrus (*r* = 0.59, *p* = 0.022, FDR-corrected), the right fusiform gyrus (*r* = 0.58, *p* = 0.024, FDR-corrected), and the left gyrus rectus (*r* = 0.60, *p* = 0.017, FDR-corrected). Furthermore, there was a significant negative correlation between pain intensity scores post-intervention and the left middle temporal pole (*r* = −0.54, *p* = 0.038, FDR-corrected). No significant association was observed between metabolism in other brain regions pain intensity at baseline and/or relative pain reduction as measured by NRS scores after SCS implantation.

## 4 Discussion

This study provides preliminary evidence for alterations in cerebral glucose metabolism in PSPS-T2 patients prior to SCS implantation, characterized by a distributed pattern of hyper- and hypometabolism across temporal, frontal, and cerebellar regions. Thereby significant hypermetabolism was observed in the left temporal pole, right inferior temporal gyrus, and bilateral cerebellar lobule VIII, whereas significant hypometabolism was identified in the right fusiform gyrus, right superior frontal gyrus, left gyrus rectus, and cerebellar vermis (lobule III). Although additional metabolic alterations were observed at exploratory thresholds, none of them survived FDR correction for multiple comparisons and should therefore be interpreted with caution. In contrast, the neurotransmitter spatial correlation analysis revealed more robust findings, including positive correlations with serotonergic receptor systems (5-HT1a, 5-HT1b, 5-HT2a, 5-HT4) as well as glutaminergic (mGluR5) distribution maps. In contrast, significant negative correlations were found with dopaminergic markers (D2, DAT, FDOPA), serotonergic 5-HT6 receptors, cholinergic α4β2 nicotinic receptors, cannabinoid CB1 receptors, kappa opioid and NMDA receptors, synaptic vesicle protein SV2A, microglial markers, COX-1, and serotonin transporters (SERT). Importantly, metabolic activity in frontal and temporal regions correlated positively with baseline pain intensity, whereas post-intervention pain intensity was shown to be negatively correlated with metabolism in the left temporal pole.

A central finding of this study is the involvement of a distributed temporo-frontal network, encompassing the temporal pole, inferior temporal gyrus, fusiform gyrus, superior frontal gyrus, and gyrus rectus. These regions are known to play a role in the integration of sensory, emotional and cognitive aspects of pain. In this light, the temporal pole has been consistently implicated in multimodal integration and affective evaluation of sensory input (Godinho et al., 2006; Herlin et al., 2021; Moulton et al., 2011; Olson et al., 2007). Converging evidence from previous studies implies that alteration in this region is associated with chronic pain states, maladaptive pain-related cognition and differences in pain sensitivity. For example, a recent study using [^18^F]FDG PET/CT in patients with chronic pain due to cancer reported increased metabolic activity in the right temporal pole and bilateral amygdala in those with poor pain control compared to those with no or well-controlled pain. Notably, this pattern was accompanied by disrupted metabolic connectivity between the amygdala and hippocampus which was interpreted as a maladaptive response to prolonged pain or stress-related amplification of pain perception (Lin et al., 2022). Moreover, a large multicenter predictive modelling study using structural MRI identified the temporal pole as contributor to interindividual differences in pain sensitivity, with higher cortical thickness being associated with lower pain sensitivity (Kotikalapudi et al., 2023). Similarly, temporal pole abnormalities have been observed in other chronic pain conditions such as knee osteoarthritis where functional connectivity changes within the limbic network were modulated by analgesic interventions (Wu et al., 2025). Collectively, these findings support the hypothesis that the temporal pole forms part of a broader temporo-limbic system involved in chronic pain processing. Interestingly, in the present study, post-intervention pain intensity correlated negatively with metabolism in the left temporal pole, suggesting that increased activity in this region may reflect a state-dependent component of ongoing pain processing that partially normalizes following treatment. While this finding may initially appear contradictory to prior findings linking temporal hypermetabolism to poorer pain control (Lin et al., 2022), this apparent difference may relate to differences in study design and clinical context. Whereas previous research focused on metabolic differences between patient groups with different pain states, the current study examined interindividual differences following intervention. To further clarify the precise functional role of the temporal pole in PSPS-T2, future studies involving larger study samples are needed.

The involvement of the inferior temporal gyrus further supports the role of temporal lobe structures in experiential and evaluative dimensions of pain. While direct evidence for the specific role of the inferior temporal gyrus remains relatively sparse, some evidence suggest that it might be part of a broader network of frontal-temporal regions involved in the processing of pain. An intracranial recording study for example demonstrated that activity in the inferior temporal gyrus, alongside superior frontal and superior temporal regions, predicts the experience of perceiving a stimulus as painful, suggesting a role in the processing of pain (Caston et al., 2023). Within this temporal system, the coexistence of hypermetabolism in the inferior temporal gyrus and hypometabolism in the fusiform gyrus suggests a functional imbalance within ventral temporal networks. This is also supported by functional connectivity studies which found altered activity in inferior temporal and fusiform regions in chronic low back pain patients (Chen et al., 2025; Gu et al., 2025). In addition to that, regional grey matter volume in the fusiform gyrus and inferior temporal area were found to be linked to pain catastrophizing, with lower grey matter volumes in these regions being associated with higher levels of catastrophizing among individuals with chronic pain (Liu et al., 2023). In the current study, fusiform metabolism correlated positively with baseline pain intensity, which further supports its relevance for the subjective experience of pain.

Next to temporal regions, frontal regions, including the superior frontal gyrus and gyrus rectus, represent key structures in the found hypometabolic network of the current study. Previously, the superior frontal gyrus has been found to be involved in the reappraisal of pain-related information which also contributes to descending inhibitory pain control pathways (Zeng et al., 2025). Functional imaging studies have revealed altered connectivity between orbitofrontal regions, including the gyrus rectus, and frontal control networks in chronic pain conditions (Li et al., 2020). Moreover, resting-state functional magnetic resonance imaging studies in adolescents have shown that greater pain frequency is associated with increased connectivity between the default mode network and the superior frontal gyrus, suggesting increased interaction between internally focused processing and cognitive control networks in individuals with higher pain burden (Jones et al., 2020). Within this framework, the hypometabolism observed in frontal regions may reflect reduced regulatory capacity, whereas the positive correlation with baseline pain intensity suggests increased, but potentially inefficient, recruitment of these regions in patients with higher pain levels.

Beyond cortical regions, the cerebellum emerges as an integral component of the observed network alterations, with hypermetabolism being observed in bilateral cerebellar lobule VIII and hypometabolism being observed in the cerebellar vermis. A recent review highlights the activation of lobule VIII and the vermis during nociceptive processing, fear conditioning, and sensory integration of pain (Manda et al., 2025). Evidence from cerebellar lesion studies suggests that even one year after cerebellar infarction, patients experienced significantly higher pain in response to acute heat and repeated mechanical stimuli compared to controls. Moreover, they experienced reduced endogenous pain inhibition and less placebo analgesia (Ruscheweyh et al., 2014). A study by Bocci et al. (2015) provides further evidence for the cerebellum's role in pain processing using transcranial cerebellar direct current stimulation (tcDCS). In healthy volunteers, cathodal stimulation induced hyperalgesia, whereas anodal stimulation produced analgesic effects and improved pain control (Bocci et al., 2015).These findings suggest that under normal conditions, the cerebellum continuously influences nociceptive processing and may thus play a central role in suppressing or filtering pain signals. In this context, the current findings showing hypermetabolism in some cerebellar regions may reflect an endogenous compensatory attempt to control pain. If the cerebellum normally inhibits pain pathways, chronic nociceptive input may force some cerebellar regions into persistent overactivity as a compensatory response. Over time, this compensation may become insufficient or maladaptive. Evidence for this idea comes from studies in which structural changes in cerebellar regions in chronic pain conditions such as migraine or fibromyalgia were found (Bilgiç et al., 2016; Kim et al., 2015; Mehnert and May, 2019) which could hint towards a maladaptive neuroplasticity in chronic pain conditions. Overall, there may thus be an over-processing of pain signals alongside an under-inhibition of pain systems in chronic pain. Further research is needed to support this assumption and to clarify the specific contribution of the cerebellum within this broader framework.

Overall, these findings may point towards a combination of enhanced nociceptive processing and reduced inhibitory control within pain-regulatory system. Further insights into the neurobiological basis of these findings are provided by the spatial correlation analysis between regional brain metabolism and neurotransmitter maps. Specifically, the positive spatial correlations with serotonergic receptor systems and mGluR5 suggest that metabolic alterations are spatially related to brain regions in which these neurochemical systems are more strongly represented. Although these normative maps do not provide a direct measure of receptor availability of function in PSPS-T2, the spatial associations are of interest in the context of the established involvement of serotonergic and glutamatergic signaling in pain processing and modulation. Serotonergic and glutamatergic systems are both centrally involved in nociceptive processing and modulation due to their widespread distribution (Chiechio, 2016; Ciranna, 2006; Liu et al., 2020). Experimental studies have demonstrated elevated spinal 5-HT concentrations, as well as sustained serotonergic signaling in neuropathic pain models (Cramer et al., 2023). Importantly, its effects are highly receptor-, region-, and context-dependent (Bardoni, 2019; Cortes-Altamirano et al., 2018; Liu et al., 2020; Viguier et al., 2013). In models of spinal cord stimulation, antagonism of spinal 5-HT2 and 5-HT1 receptors abolished the antinociceptive effects of SCS, indicating that descending serotonergic pathways are required for SCS-mediated analgesia (Tazawa et al., 2015). Additional studies further demonstrated that activation of spinal 5-HT2a, 5-HT3, and 5-HT4 receptors contributes to the analgesic effects of SCS (Song et al., 2011) suggesting a role in central sensitization as well as regulation of neuronal excitability. The simultaneous negative correlation with serotonin transporter (SERT) alongside positive correlations with multiple serotonergic receptors may suggest that different components of the serotonergic system have distinct spatial relationships with the metabolic pattern. Together, these findings support the relevance of serotonergic signaling in pain modulation and imply that the metabolic pattern does not map uniformly onto the serotonergic system but rather shows component specific spatial relationship.

Another relevant finding concerns the observed positive correlation with mGluR5. Glutamatergic signaling plays a central role in central sensitization and the amplification of nociceptive input (Latremoliere and Woolf, 2009; Xie et al., 2023). Interactions between serotonergic and glutamatergic systems are well established, with evidence indicating that 5-HT receptor activation can modulate glutamatergic neurotransmission (Yuan et al., 2022). Notably, research has demonstrated that combined antagonism of 5-HT2A and mGluR5 produces enhanced anti-nociceptive effects compared to targeting either system alone, suggesting a functional coupling between these pathways in the regulation of pain (Choi et al., 2025). The observed mGlu5 association is therefore consistent with the established involvement of glutamatergic signaling in pain-related processing and further supports its relevance to the observed metabolic pattern.

In contrast, the negative correlations with dopaminergic markers, cannabinoid and opioid receptors, as well as synaptic (SV2A) and neuroimmune (TSPO, COX-1) markers indicate an inverse spatial relationship between the observed metabolic pattern and the normative distribution of these markers. Dopaminergic pathways, particularly within frontostriatal circuits, are known to modulate the motivational and affective dimensions of pain (Van Roosendaal et al., 2025), while opioid and cannabinoid systems are central to endogenous pain inhibition (Nadal et al., 2013; Secondulfo et al., 2024). Similarly, SV2A and TSPO are markers of synaptic integrity and immune activation, respectively (Bavarsad and Grinberg, 2024; Che et al., 2025; Sun et al., 2020). The relative lack of spatial overlap with these systems therefore suggests metabolic alterations identified in PSPS-T2 are particularly aligned with the regional distribution of serotonergic and glutamatergic systems compared to the other molecular systems examined.

Overall, the spatial correlation analysis suggest that the metabolic alterations observed in PSPS-T2 are differentially related to the regional distribution of several neurotransmitter and molecular systems. The observed alterations across temporal, frontal, and cerebellar regions point to a distributed pattern of metabolic changes involving regions associated with sensory, affective, and cognitive dimensions of pain processing. Converging evidence from structural, functional and intracranial studies supports the involvement of these regions in chronic pain, thereby reinforcing the validity of the current findings. The associated serotonergic and glutamatergic signatures further suggest that these network-level alterations may be related to the spatial organization of neurotransmitter systems with established roles in pain processing and modulation. Rather than reflecting isolated regional abnormalities, the results support the concept of chronic pain as a disorder of large-scale brain network organization arising from an imbalance between excitatory processing and modulatory control mechanisms.

This study is the first to investigate the brain metabolism of PSPS-T2 patients prior to SCS intervention. A key strength of this study is the use of a dedicated [^18^F]FDG PET neuro-imaging protocol in a prospective setting. The dedicated [^18^F]FDG PET neuro-imaging protocol is well-established and thereby has direct clinical applicability. By combining group-level SPM analysis, and neurotransmitter-informed spatial correlation (JuSpace), we ensured consistency across independent analytical approaches. To our knowledge, this is the first study investigating cerebral metabolism in PSPS-T2 patients prior to SCS implantation using this multi-tiered approach. Nevertheless, several limitations should be acknowledged. The sample size was relatively small, which limits statistical power and reinforces the exploratory nature of the findings. Whole-brain activity normalization was applied for PET normalization, although widespread cortical and subcortical changes in metabolism were observed in this study. Still, the use of whole-brain normalization was deemed the most appropriate normalization method in this population as widespread (structural) alterations in cortical and subcortical metabolism have been reported in chronic low back pain and no regions have robustly been reported to be spared (for a recent review, see (Li et al., 2024)). Within this context of diffuse brain diseases, whole-brain normalization is considered the most robust and least assumption-driven approach (Cerne et al., 2025). Consequently, whole-brain normalization is also used in PET neuroimaging of neurodegenerative dementias. One limitation concerns the fact that no post-treatment PET scans were acquired, leaving it unclear whether the observed metabolic abnormalities are state-dependent and reversible following SCS treatment. Longitudinal imaging studies with repeated [^18^F]FDG PET before and after SCS could provide further mechanistic insight and help determine whether metabolic changes are a prerequisite or a by-product of symptom relief. Furthermore, stimulation parameters and SCS program use varied between patients and could not be standardized or fully accounted for, reflecting the individualized nature of SCS treatment in clinical practice. Therefore, a potential confounding effect of differences in SCS settings on cerebral metabolism cannot be excluded. In addition, in this study only semi-quantitative PET metrics (SUVR) were used rather than fully quantitative kinetic modelling which limits the possibility of detecting and accounting for potential differences in tracer delivery or metabolic rate. Future studies could profit from incorporating kinetics methods such as arterial sampling to allow for a more quantitative modelling and more precise estimates of regional cerebral glucose metabolism. Consequently, this approach has the potential to support the development of more objective measures in chronic pain assessment. Additionally, as no subject-specific MRI was acquired, spatial normalization was performed using direct PET-to-template registration. Although this approach is commonly applied in FDG PET studies and allows robust group-level analyses, the absence of an anatomical MRI may introduce minor registration inaccuracies and limits anatomical precision. Finally, even though patients underwent psychological screening as part of the SCS treatment pathway, the potential contribution of subclinical psychological symptoms, such as anxiety or depression, to the observed metabolic alterations cannot be completely excluded. Future studies should therefore consider including standardized measures of psychological symptoms to further assess their potential contribution to the observed metabolic alterations.

## 5 Conclusion

This study provides preliminary evidence for distributed alterations in cerebral glucose metabolism involving temporo-frontal and cerebellar regions that play a role in the sensory, emotional and cognitive aspects of pain processing. The accompanying neurotransmitter spatial correlations suggest a particular involvement of serotonergic and glutamatergic systems, supporting the concept of PSPS-T2 as a disorder of large-scale brain network dysregulation rather than isolated regional abnormalities. Although limited by the small simple size, these findings offer novel insights into the neurobiological mechanisms underlying PSPS-T2. Future longitudinal studies with larger cohorts and post-treatment imaging are needed to validate the current findings.

## CRediT authorship contribution statement

**Lara S. Burmeister:** Writing – review & editing, Writing – original draft, Visualization, Validation, Methodology, Investigation, Data curation, Conceptualization. **Max van Grinsven:** Writing – review & editing, Visualization, Formal analysis, Data curation. **Bert-Kristian van Roosendaal:** Writing – review & editing, Conceptualization. **Richard L. Witkam:** Writing – review & editing, Validation, Supervision, Methodology, Conceptualization. **Inge Arnts:** Writing – review & editing, Resources, Investigation. **Stephanie Janssen:** Writing – review & editing, Resources, Investigation. **Jessica Wegener:** Writing – review & editing, Resources, Investigation. **Kris C.P. Vissers:** Writing – review & editing, Validation, Supervision, Methodology, Conceptualization. **Anne I.J. Arens:** Writing – review & editing, Formal analysis, Data curation. **Martin Gotthardt:** Writing – review & editing, Validation, Supervision, Methodology, Conceptualization. **Michael Rullmann:** Writing – review & editing, Visualization, Validation, Software, Formal analysis, Data curation. **Dylan Henssen:** Writing – review & editing, Validation, Supervision, Methodology, Funding acquisition, Formal analysis, Data curation, Conceptualization.

## Consent to participate

Written informed consent was obtained from all subjects.

## Ethics approval

The study was conducted according to the guidelines of the Declaration of Helsinki. The medical research ethics committee of the Radboud University Medical Center (dossier number ‘2021–13293’) approved the study protocol. All patients provided written informed consent. Clinical trial number not applicable.

## Ethics declaration

Written informed consent to take part in the study and to publish the article has been obtained from all participants or their legal representatives. The privacy rights of participants have been observed.

This study was performed in compliance with relevant laws, regulatory frameworks and guidelines where the research took place. This study was approved by the METC Oost-Nederland. (Approval No. 2021–13,293)

## Funding

No funding was received towards this work.

## Declaration of competing interest

The authors declare the following financial interests/personal relationships which may be considered as potential competing interests: Dr. Henssen is supported by the Clinician Scientist Program of Universitätsmedizin Leipzig, which facilitates the integration of clinical practice and scientific research. If there are other authors, they declare that they have no known competing financial interests or personal relationships that could have appeared to influence the work reported in this paper.

## Data Availability

The derived group-level statistical T-maps underlying the neuroimaging analyses are openly available through NeuroVault (https://neurovault.org/collections/24569/).
